# Mobile.net: Mobile Telephone Text Messages to Encourage Adherence to Medication and to Follow up With People With Psychosis: Methods and Protocol for a Multicenter Randomized Controlled Two-Armed Trial

**DOI:** 10.2196/resprot.2136

**Published:** 2012-08-02

**Authors:** Maritta Välimäki, Heli Hätönen, Clive E Adams

**Affiliations:** 1 Department of Nursing ScienceUniversity of Turku and Southwest Hospital DistrictTurkuFinland; 2 Department of Nursing ScienceUniversity of TurkuTurkuFinland; 3 Institute of Mental HealthDivision of PsychiatryUniversity of NottinghamNottinghamUnited Kingdom

**Keywords:** Adherence, text messages, psychosis

## Abstract

**Background:**

Schizophrenia is a high-cost, chronic, serious mental illness. There is a clear need to improve treatments and expand access to care for persons with schizophrenia, but simple, tailored interventions are missing.

**Objective:**

To evaluate the impact of tailored mobile telephone text messages to encourage adherence to medication and to follow up with people with psychosis at 12 months.

**Methods:**

Mobile.Net is a pragmatic randomized trial with inpatient psychiatric wards allocated to two parallel arms. The trial will include 24 sites and 45 psychiatric hospital wards providing inpatient care in Finland. The participants will be adult patients aged 18–65 years, of either sex, with antipsychotic medication (Anatomical Therapeutic Chemical classification 2011) on discharge from a psychiatric hospital, who have a mobile phone, are able to use the Finnish language, and are able to give written informed consent to participate in the study. The intervention group will receive semiautomatic system (short message service [SMS]) messages after they have been discharged from the psychiatric hospital. Patients will choose the form, content, timing, and frequency of the SMS messages related to their medication, keeping appointments, and other daily care. SMS messages will continue to the end of the study period (12 months) or until participants no longer want to receive the messages. Patients will be encouraged to contact researchers if they feel that they need to adjust the message in any way. At all times, both groups will receive usual care at the discretion of their team (psychiatry and nursing). The primary outcomes are service use and healthy days by 12 months based on routine data (admission to a psychiatric hospital, time to next hospitalization, time in hospital during this year, and healthy days). The secondary outcomes are service use, coercive measures, medication, adverse events, satisfaction with care, the intervention, and the trial, social functioning, and economic factors. Data will be collected 12 months after baseline. The outcomes are based on the national health registers and patients’ subjective evaluations. The primary analysis will be by intention-to-treat.

**Trial Registration:**

International Standard Randomised Controlled Trial Number (ISRCTN): 27704027; http://www.controlled-trials.com/ISRCTN27704027 (Archived by WebCite at http://www.webcitation.org/69FkM4vcq)

## Introduction

Schizophrenia is a high-cost, chronic, serious mental illness [1]. The global prevalence of schizophrenia is about 1%-1.5% [2]. Among mental, neurological, and substance use disorders, schizophrenia is the third highest in the ranking of global burden in disability-adjusted life years [3]. Studies have shown that a prevalence among relapsed patients varies, for example from 52% [4] to 80% within 5 years [5]. Nonadherence to antipsychotic medication is highly prevalent, has a deleterious impact on the course of the illness [6], and is the single most important predictor of relapse and readmission [7], with all the subsequent costs being an additional burden [8-11]. People may be nonadherent to prescribed medications for many reasons. Forgetting to take the medication, feeling that it is unnecessary, and disliking adverse effects are all common [12]. In any event, it is probably an underestimate of real life that over half of people with schizophrenia stop medication within 1 year of discharge from hospital [13]. Individually tailored approaches to encourage adherence to medication have been called for [6].

Although simple and direct telephone calls [14-17] and well-timed prompting letters [18] may decrease nonattendance at psychiatric outpatient departments, short message service (SMS) with mobile phones may provide better access to a population group. Text messaging is common, inexpensive, and less intrusive than a phone call. It is also possible to send preprogrammed batches of messages. A trust-wide survey in the United Kingdom with 141 psychiatric inpatients (100 patients with psychotic illness and 41 who had a diagnosis of a nonpsychotic illness) showed that 55% had a mobile phone, 56% were able to use text messages, and 76% were willing to receive text messages from the National Health Service foundation trust [19]. We know of no similar data in Finland. In 2011, however, 98% of Finnish households had mobile phones, and about 20% had more than one mobile phone [20].

While there is a clear need to improve treatment and expand access to care for people with schizophrenia, mobile technologies could be developed to support this area [3]. As preparation for this trial, we are completing a Cochrane review [21]. We have identified relevant important pioneering work that has been reported in international conferences [22,23] (n = 865). There are already some applications in other areas of heath care. SMS has been used in asthma care [24], smoking cessation [25], promoting safer sex and sun safety in young people [26], and monitoring and coaching persons with chronic migraine [27]. People with schizophrenia or similar illnesses are, however, different from the standard health service population. SMS, if tailored and used sensitively, has the potential to be both potent and possible to implement in everyday care. To justify this, however, and not simply impose one more unevaluated intrusion into the lives of people with these disturbing illnesses, high-grade adequately powered data from randomized trials are required [6].

The Mobile.Net study will establish the impact of tailored mobile telephone text messages to encourage adherence to medication and to follow up with people with psychosis at 12 months. Mobile.Net is a short name for the trial to help health care professionals and participants be aware of the project.

## Methods

### Design

Mobile.Net is a multicenter, randomized controlled study with a two-armed trial conducted in Finland (24 sites and 45 hospital wards).

### Hypothesis

We hypothesized that tailored mobile telephone text messages acceptable to the individual patient would reduce use of services by people with serious mental illness whose previous use of services has been high.

### Inclusion and Exclusion Criteria

Inclusion criteria are patients aged 18-65 years, of either sex (Table 1), with antipsychotic medication [28] on discharge from a psychiatric hospital, who have a mobile phone, are able to use the Finnish language, and are able to give written informed consent to participate. We will include no formal test of capacity but will rely on the judgment of experienced health care professionals in their routine assessment, when nearing the point of discharge, of the patients’ understanding, retention, assimilation, and communication of all information, including that relevant to the study. Further formalized assessment is not part of routine care.

Patients who have planned a nonacute treatment period or are being treated in forensic psychiatric services will be excluded.

**Table 1:** Dummy table for background characteristics in intervention (short message service) and control groups at baseline.

Characteristic		Intervention		Control	
		n	%	n	%
Age (years)		DUMMY TABLE - CELLS INTENTIONALLY EMPTY			
**Sex**					
	Male				
	Female				
	Other				
**Marital status**					
	Single				
	Married				
	Divorced				
	Widowed				
**Vocational education**					
	None				
	Vocational training courses				
	Primary vocational skill certificate				
	Secondary vocational skill certificate				
	University degree				
**Employment status**					
	Employed				
	Retired				
	Self-employed				
	Student				
	Job seeker				
**Number of previous psychiatric treatment periods**					
	1				
	2 or more				
Age at first contact with psychiatric services (years)					

### Interventions

#### Intervention Group

The intervention group will receive semiautomatic SMS messages after their discharge from psychiatric inpatient care. The intervention is patient led rather than researcher led to increase acceptability of the prompt. Therefore, during the discharge process from psychiatric hospital care, patients will choose text message content areas related to their medication and follow-up appointments. Additionally, they will be able to choose messages related to other daily issues (eg, hygiene, physical exercise, nutrition, daily routines, clothing, safety, communication, taking care of pets, following rules, hobbies, work or other activities, household tasks, symptom management, or other supporting messages). Timing, frequency, and conditions under which interventions will be withheld will be decided by the patient. The content of these messages had been designed by patients in a consumer association, and by health care professionals in hospital wards and outpatient clinics. The SMS messages will continue for the duration of the study period (12 months) or until participants no longer want to receive the messages. We do not envisaged that the intervention will interfere in any way with routine outpatient care. Patients will be encouraged to contact the researchers or health care staff if they feel that they need any adjustment of the message.

#### Control Group

Patients in the control group will receive standard treatment care in an outpatient unit based on the existing system in Finland.

All patients will receive usual care at the discretion of their team (psychiatry and nursing).

### Randomization and Masking

The randomization codes (permuted block design with 4 patients per block) were computer generated by an independent statistician. Others, completely independent of the trial team, inserted these numbers into sealed envelopes. While this is a multicenter study to be run in 24 sites—that is, health service organizations (and 45 study wards) with psychiatric beds in Finland—patients within each ward will be randomly allocated separately. The is an open-label study.

Written allocation of assignment will be sealed in individual envelopes marked with study identification numbers, which will be distributed to all study wards. Research nurses on study wards will sequentially assign sealed envelopes in a predetermined order to patients who fulfill the inclusion criteria and give their written informed consent during their discharge process. Investigators will be masked to data until the statistician releases the database, although a data management committee will undertake ongoing safety surveillance. Study participants and staff will not be masked; this would not reflect real-world care. However, randomization and analyses will be undertaken by investigators masked to treatment allocation.

The sealed envelopes with study identification numbers will be opened by a research nurse or a patient in an ascending manner. Patients will be allocated to the intervention or control group.

### Allocation Concealment

Participants and the investigators enrolling the participants will not foresee assignment. We will use numbered sealed envelopes in different data collection organizations. Whereas patients and health care staff will be aware of the allocated arm, outcome assessors and data analysts will be kept blinded to the allocation.

### Primary and Secondary End Points

The primary outcomes are service use and healthy days by 12 months based on routine data (admission to psychiatric hospital, time to next hospitalization, time in hospital during this year, and healthy days). The secondary outcomes are service use, coercive incidents, medication, adverse event, satisfaction with care, intervention, and the trial, social functioning, and economic factors. M1 referral is a referral for observation by any physician if he or she considers it likely that criteria for involuntary admission are fulfilled. This sets in motion the process by which the patient is later examined by a second doctor in a psychiatric hospital. This doctor must be a psychiatrist. At this stage, the patient can be admitted voluntarily or involuntarily, or discharged [29]. Data will be collected 12 months after baseline (see Table 2 [30,31]).

**Table 2:** Dummy table for primary and secondary outcomes and end points.

Outcome				Intervention				Control			
				n (%) or mean (SD)		RR^a^ (95% CI^b^)	*P *value	n (%) or mean (SD)		RR (95% CI)	*P *value
**Primary outcome**				DUMMY TABLE - CELLS INTENTIONALLY EMPTY							
	**Service use/healthy days**										
		Admission to psychiatric hospital									
		Time to next hospitalization (days)									
		Time in hospital during this year (days)									
		Healthy time (days)									
**Secondary outcomes**											
	**Service use**										
		Type of admission									
			M1 referral^c^								
			Mental examination								
			Determination of treatment								
			Other								
		Involuntary treatment									
		General hospital treatment									
		Use of private care									
		Length of involuntary psychiatric treatment (days)									
		Length of general hospital stay (days)									
	**Coercion**										
		Coercive incidence									
		Type of coercive incidence									
			Seclusion								
			Physical restraint								
			Intramuscular medication								
			De-escalation								
	**Medication**										
		Type of medication									
			Antipsychotic								
			Antipsychotic + antidepressant								
		Medication									
	**Adverse event**										
		Any (yes)									
		Death (yes)									
	**Satisfaction with care/intervention/trial**										
		Satisfied with care									
		Requested to stop SMS^d^									
		Left the study early									
	**Social functioning**										
		Quality of life									
		Disability support									
	**Economic factors**										
		Direct cost (€)									
		Indirect cost (€)									

^a ^Relative risk.

^b ^Confidence interval.

^c ^Referral for observation.

^d ^Short message service.

The outcomes will be based on register data [30,31] and patients’ subjective evaluations.

### Adverse Event Reporting

Safety assessments will include all adverse or serious adverse events, and subjective symptoms reported by clinical staff or study personnel, participants, or relatives. Adverse event monitoring data will be collected by the following methods: (1) reports from clinical staff or study personnel, and (2) text message, mailing, phone call, or survey after 12 months during the 1-year intervention with the question “Have you experienced any new and serious health problems since you enrolled in the Mobile.Net Study? If yes, please describe them.”

We will provide categories of possible adverse events. Adverse events will be categorized as severe if they are life-threatening or fatal, require or prolong a hospitalization, or result in a major disability. In addition, adverse event may be categorized as unexpected or expected (Table 3).

**Table 3:** Dummy table for number of adverse events.

		Intervention	Control
**Expected severe adverse events**		DUMMY TABLE - CELLS INTENTIONALLY EMPTY	
	Life-threatening or fatal		
	Requiring or prolonging a hospitalization		
	Resulting in a major disability		
**Unexpected severe adverse events**			
	Life-threatening or fatal		
	Requiring or prolonging a hospitalization		
	Resulting in a major disability		
**Expected adverse events**			
	Medical		
	Psychiatric		
	Substance use		
**Unexpected adverse events**			
	Medical		
	Psychiatric		
	Substance use		

The potential safety concerns about possible psychiatric effects of exposure to a mobile phone have been discussed for more than two decades [32], but published results are still inconsistent and inconclusive [33-35]. As the study intervention is a communication system and provides a method of supporting patients’ adherence in regular mental health services, we do not expect serious adverse events as a result of the intervention. We will analyze treatment-emergent adverse events, defined as all adverse and serious adverse events occurring between randomization and when the patient completes the study.

### Statistical Analysis Plans

#### Sample Size and Power Calculations

The study has been powered for the primary outcome measure. We have systematically searched for but not found any directly relevant published work. In London, for example, 65% of potentially eligible people had been admitted to the hospital in the past 12 months [36]. Our aim in Finland will be to reduce use of acute care by at least five percentage points (a relative risk of 0.92). To do this with 80% power at a 5% 2-sided significance level, we would require 1511 participants in each of the two arms (Stata v10; StataCorp LP, College Station, TX, USA).

#### Type of Analysis and Missing Data

All analyses will be based on the intention-to-treat principle. For incomplete (missing) data, we will use multiple imputation by chained equations, which makes appropriate assumptions based on the predictors of outcome and predictors of loss to follow-up. Missing outcome data will be evaluated where they are balanced in numbers across intervention groups and with similar reasons across control groups. The same procedures will be used for secondary outcomes.

#### Statistical Tests

The primary outcome will be relative risk of admission to psychiatric inpatient services (Table 2). Secondary outcomes will involve calculation of relative risk or mean differences and their corresponding 95% confidence intervals. To evaluate the clinical outcomes of the study, we will use both descriptive and inferential statistics. Descriptive statistics will be used to evaluate outcomes at the end point and differences between individuals and groups by exploratory analyses (chi-square tests, *t *test or Mann-Whitney *U *test, and nonparametric bootstrap methods for skewed cost data). The principal analysis will compare the primary and secondary outcome measures at 12 months comparing for baseline (preintervention) measures using analysis of covariance.

### Subgroup Analysis Planned

We will analyze subgroups for the primary outcome of people with schizophrenia-like illness compared with others who need antipsychotics. The direct and indirect costs of implementing this service will be examined through the prospective collection of staff time and resource requirements. Total costs of implementing the service will be estimated through local salary and overhead costs and by reference to nationally agreed-upon and unit costs. The cost effectiveness of the approach will be calculated through incremental cost-effectiveness ratios of costs per nonattendance avoided.

### Ethics Issues

The Ethics Committee of the Hospital District of Southwest Finland approved the study on December 16, 2010 (ETMK 109/180/2010). Any regulations addressing the conduct of trials involving vulnerable populations and clinical trials investigating products will be taken into account [37].

We did will not use any formal tests of capacity to evaluate patient competence. We will rely on the judgment of experienced health care professionals in their routine assessment of patients’ status and well-being. Study participants and staff will not be masked (blinded), so as to reflect real-world care. This may, however, cause ethical and practical outcomes. Participants may respond better if they know they have received a promising new treatment or worse if they received only standard care. Nurses may motivate patients more toward a specific group depending on which group (intervention or control) nurses prefer [38].

### Informed Consent Form and Information Sheet

All participants will give their consent to participate on the basis of appropriate information and with adequate time to consider this information and to ask questions [39]. We will obtain written consent to participate in the study through an informed consent form evaluated by the Ethics Committee of The Hospital District of Southwest Finland. Patients will be invited to sign the consent form after they have received oral and written information about the study. Patients will receive two type of written information material: (1) a short, 1-page information leaflet about the study to help patients orient themselves to the study, and (2) later, near the discharge process, more detailed written information describing the study and its phases. Participants will be made aware, before consenting, that they are free to withdraw without obligation at any time and that such an action will not adversely affect any aspect of their care.

### Independent Data Safety and Monitoring Committee

The members of the independent data safety and monitoring committee will consist of two experts in mental health care, a representative of a patient association, and a statistician.

### Interim Analysis and Stopping Rules

An independent data safety and monitoring committee will be established. The trial statistician will carry out analyses, blinded to allocation, and report the results to the data safety and monitoring committee. Together with an independent statistician, the data safety and monitoring committee will review efficacy and safety data.

Due to the nature of the data collated on the national register, it is not possible to conduct an interim analysis after the recruitment has started. The information in the register data will be available to the researchers after 1 calendar year (ie, data have a 1-year lag time). However, adverse events, early dropout, nurses’ opinions, and patients’ experiences will be monitored and analyzed throughout the study.

### Indemnities

The Finnish national patient insurance system covers patients’ possible loss and compensates them for that loss based on the Patient Injuries Act [39] (585/1986; amendments up to 1100/2005 included). This insurance is held by professions in health care or medical care to compensate for harm caused to patients through accident or neglect. This insurance covers harm caused by treatment, infection, accident, equipment, facilities, or installations.

### Publication Plan

We plan one major results paper authored by M Välimäki, H Hätönen, CE Adams, and a collective (Finland’s Mobile.Net Collaborative Group) comprising all active collaborators, and published in parallel with a relevant Cochrane review. We propose making data available for prospective meta-analysis.

### Funders

Grant support for the intervention was received from the Academy of Finland (132581), Satakunta Hospital District, State Subsidy System (EVO) (12/2010, 81096), and the Hospital District of Southwest Finland, EVO (13893).

### Role of the Funding Source

The funders do not have any role in study design, data collection, analysis, decision to publish, interpretation, or preparation of the manuscript. The corresponding author will have full access to all the data in the study and will have final responsibility for the decision to submit for publication.

### Recruitment Schedule

Recruitment started in November 2011 and will finish December 2012. Results will be reported in 2015.

## Abbreviations

SMS: short message service
